# Advancing MSC-EV Therapies: Harnessing Preconditioning and Mito-EVs to Tackle Neuroinflammation and Neurodegeneration

**DOI:** 10.3390/pharmaceutics18060730

**Published:** 2026-06-12

**Authors:** Eva Costanzi, Luca Fontana, Francesca Giroldo, Silvia Coco

**Affiliations:** 1 School of Medicine and Surgery, University of Milano-Bicocca, 20900 Monza, Italy; l.fontana12@campus.unimib.it (L.F.); f.giroldo@campus.unimib.it (F.G.); 2 Ph.D. Program in Neuroscience, School of Medicine and Surgery, University of Milano-Bicocca, 20900 Monza, Italy

**Keywords:** mesenchymal stromal/stem cells, extracellular vesicles, mitochondrial EVs, preconditioning, neuroinflammation, neurodegeneration, immunomodulation

## Abstract

Neuroinflammation plays a central role in the onset and progression of neurodegenerative disorders. Several disease-modifying therapies have been developed to target neuroinflammatory pathways in specific disorders. However, their ability to stop disease progression or restore neuronal and mitochondrial homeostasis remains limited. This is still a major unmet clinical need. In this context, mesenchymal stromal cell (MSC)-derived Extracellular Vesicles (EVs) have emerged as a promising cell-free therapeutic strategy due to their ability to modulate immune responses and promote neuroprotection through the delivery of bioactive cargo. Recent evidence has identified a distinct subset of EVs, known as mitochondrial EVs (mito-EVs), which carry mitochondrial DNA, proteins, and functional components. These vesicles may uniquely influence cellular bioenergetics, redox balance, and neuroinflammatory signaling, offering additional therapeutic potential compared to conventional MSC-EVs. This review summarizes the role of MSC-derived EVs in neuroinflammatory disorders, with a particular focus on mito-EVs. It also discusses preconditioning strategies to enhance EV efficacy, including hypoxic, inflammatory, pharmacological priming and genetic engineering approaches. Finally, we critically evaluate current preclinical evidence regarding the treatment of major neurodegenerative disorders, including Alzheimer’s disease, Parkinson’s disease, Multiple Sclerosis, and Amyotrophic Lateral Sclerosis, as well as Traumatic Injury, highlighting the key challenges for clinical translation.

## 1. Introduction

Over the past decade, neuroinflammation has emerged as a central component in the pathogenesis of major neurodegenerative disorders. Once considered mainly as a secondary response to neuronal injury, inflammatory processes within the central nervous system (CNS) are now recognized as active contributors to disease onset and progression [1]. This shift in perspective has stimulated intense research efforts aimed at identifying therapeutic strategies capable of modulating neuroinflammatory pathways [2]. In this context, mesenchymal stromal/stem cells (MSCs) have gained considerable attention due to their immunomodulatory and neuroprotective properties. Notably, their therapeutic effects are largely mediated by paracrine mechanisms, particularly via their secretome including the release of Extracellular Vesicles (EVs). EVs are lipid bilayer particles involved in intercellular communication, transferring proteins, lipids, and nucleic acids that reflect the state of the originating cell. They are mainly classified into exosomes (Exos) and microvesicles (MVs) based on their biogenesis [3]. However, current guidelines recommend classification based on physical and molecular features due to technical limitations in distinguishing subtypes. Accordingly, in line with MISEV 2023 recommendations, the term “EVs” is used throughout this review to encompass heterogeneous vesicle populations [3].

Among emerging approaches, MSC-derived EVs (MSC-EVs) have attracted growing interest as cell-free alternatives to MSC-based therapies. MSC-EVs can transfer bioactive molecules and modulate both immune and neuronal responses, promoting neuroprotection while reducing the risks associated with cell-based therapies [4,5,6].

Furthermore, although scientific evidence is still limited, another innovative approach involves the use of mitochondrial Extracellular Vesicles (mitovesicles or mito-EVs), small vesicles derived from mitochondria that preserve mitochondrial inner structures. Mito-EVs act as important indicators of cellular metabolic state and stress conditions, and they may offer several therapeutic advantages by helping restore neuronal energy balance and reduce oxidative stress [7].

### 1.1. Aim of the Review

Despite significant advances in understanding neuroinflammatory mechanisms, effective disease-modifying therapies for neurodegenerative disorders remain limited. This unmet clinical need has prompted the exploration of alternative strategies capable of targeting inflammation while maintaining neuronal integrity.

MSC-EVs are increasingly recognized as key immunomodulatory mediators in neurodegenerative disorders, exerting both anti-inflammatory and neuroprotective effects [5]. In parallel, mitochondrial dysfunction has emerged as a key driver of both neuroinflammation and neurodegeneration. In this context, growing evidence supports the existence of a distinct subpopulation of EVs, mito-EVs, which may offer additional therapeutic advantages. Mito-EVs carry mitochondrial DNA, proteins, and functional components and have been detected in the brain under both physiological and pathological conditions [8]. Their cargo can be dynamically modulated during neurodegenerative progression, with implications for neuronal bioenergetics, redox balance, synaptic plasticity and inflammatory signaling [9]. By transferring functional mitochondrial components to stressed neurons or glial cells, MSC-derived mito-EVs may restore energy metabolism, reduce oxidative stress and modulate inflammatory responses, effects that extend beyond those achieved by conventional MSC-EVs alone.

The primary aim of this review is to highlight the therapeutic potential of MSC-EVs in neuroinflammatory disorders, with particular emphasis on mito-EVs [10] and on strategies to modulate EV cargo through preconditioning approaches, aspects that have been only marginally addressed in the previous literature. In particular, this review integrates emerging evidence on mito-EVs with neuroinflammatory mechanisms [11,12] and discusses approaches to enhance MSC-EV efficacy through hypoxic, inflammatory, and pharmacological priming, which have been shown to reshape EV cargo and improve functional outcomes in preclinical models [13].

Finally, this review summarizes and critically evaluates current evidence on MSC-EVs in experimental models of neuroinflammation, with a focus on Alzheimer’s disease (AD), and emerging data in Parkinson’s disease (PD), Multiple Sclerosis (MS) and Amyotrophic Lateral Sclerosis (ALS), as well as Traumatic Injury, and will outline current limitations and future directions toward clinical translation. Notably, the existing literature is more extensive for AD, with comparatively fewer studies on PD and MS; however, MSC-EVs demonstrate broader therapeutic potential across neuroinflammatory disorders [14,15]. The articles cited in this review were selected based on their relevance to the topic, originality, scientific quality, and publication date, focusing on literature from the last decade (2015–2026).

### 1.2. Overview of Neuroinflammation

Neuroinflammation refers to the immune response of the CNS, which is mediated by diverse types of resident glial cells including dendritic cells, pericytes, microglia and astrocytes. Under physiological conditions, this tightly regulated response plays a crucial role in maintaining CNS homeostasis by clearing apoptotic debris, supporting synaptic remodelling and facilitating tissue repair following injury or stress. However, when neuroinflammatory signalling becomes chronic or dysregulated, phagocytic activity and the efficacy of microglia in maintaining CNS homeostasis decrease, favouring the shift from a protective to a detrimental process, contributing to neuronal dysfunction and degeneration [1].

At the molecular level, neuroinflammation is driven by the release of pro-inflammatory cytokines, including IL-1β, TNF-α, and IL-6, as well as by damage-associated and pathogen-associated molecular patterns (DAMPs and PAMPs) released from injured or stressed cells. These signals are detected by innate immune receptors, such as toll-like receptors (TLRs), expressed on microglia, leading to the activation of downstream inflammatory pathways. Among these, NF-kB and the NLRP3 inflammasome play critical roles, promoting sustained cytokine production, reactive oxygen species (ROS), and amplification of the inflammatory response.

Microglia, the resident immune cells of the CNS, serve a critical role as the brain’s surveillance and in the regulation of the neuroinflammatory response due to their ability to recognize danger signals through pattern recognition receptors (PRRs) and actively remove abnormal extracellular protein aggregates to maintain CNS homeostasis. In fact, microglia display remarkable functional plasticity, because depending on the microenvironment they undergo morphological and molecular changes: they can adopt pro-inflammatory and potentially neurotoxic states, which are characterized by a phagocytic and ameboid phenotype (often referred to as M1-like), or shift toward anti-inflammatory and neuroprotective phenotypes, which are characterized by a highly branched phenotype (M2-like) [16]. Importantly, these phenotypes do not represent fixed states but rather a dynamic spectrum that is continuously shaped by interactions with astrocytes, neurons and infiltrating immune cells [17].

Astrocytes also play a critical and active role in neuroinflammation, constituting an important downstream aspect of neuroimmune regulation. Upon activation, they undergo profound phenotypic and functional changes, often referred to as reactive astrogliosis. Reactive astrocytes can acquire either neurotoxic or neuroprotective profiles (commonly described as A1 and A2 phenotypes). These distinct states contribute to synaptic dysfunction, BBB alterations, and modulation of inflammatory signaling. Astrocyte activation is tightly linked to microglial responses, as microglia-derived cytokines can induce astrocytic reactivity, further amplifying the inflammatory cascade [18,19].

Additionally, under pathological conditions, peripheral immune cells can infiltrate the CNS due to the disruption of BBB integrity, which is normally composed of pericytes, astrocytes, and brain endothelial cells and maintains CNS stability by regulating the passage of molecules across it. Circulating monocytes, macrophages, and lymphocytes contribute to the inflammatory milieu through cytokine release, antigen presentation, and interaction with resident glial cells. This crosstalk between central and peripheral immunity further increases the complexity of neuroinflammatory processes [20,21].

A key feature of neuroinflammation is its bidirectional relationship with neurodegeneration. Neuronal injury and protein aggregation promote glial activation through the release of DAMPs and oxidative stress signals. In turn, sustained inflammatory activation exacerbates neuronal damage and mitochondrial dysfunction, reinforcing degenerative processes through sustained engagement of pathways such as NF-kB and NLRP3 [1]. Specifically, NLRP3 acts as a sensor of DAMPs. After NF-κB-mediated priming, NLRP3 oligomerizes and recruits ASC and procaspase-1. This leads to the formation of the inflammasome complex, which strengthens the inflammatory response and contributes to disease progression and functional impairment [22].

Persistent neuroinflammation is closely associated with mitochondrial dysfunction. Impaired mitochondrial activity leads to reduced ATP production, altered energy homeostasis, and increased ROS generation, all of which contribute to neuronal injury. In parallel, chronic activation of microglia is associated with impaired phagocytic capacity and sustained inflammatory signaling [23].

Collectively, these mechanisms identify neuroinflammation as a key therapeutic target for limiting neuronal damage and slowing disease progression. In the following section, we will examine how these processes contribute to the pathogenesis of major neurodegenerative diseases, highlighting both shared pathways and disease-specific features.

### 1.3. Neuroinflammation Across Major Neurodegenerative Diseases

The CNS is one of the most energy-demanding organs in the body and it is traditionally considered an immune-privileged site. Immune surveillance within the CNS is mainly mediated by resident immune cells, including microglia and other CNS-associated macrophages, along with a small population of dendritic cells distributed throughout the brain and spinal cord [24]. Among these, microglia play a key role in maintaining tissue homeostasis and responding to pathological stimuli.

**Figure 1:**
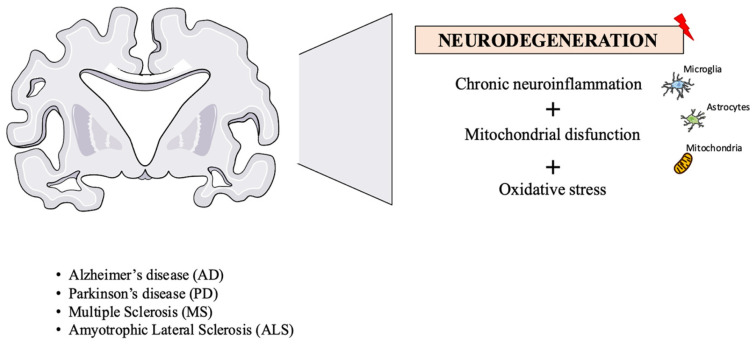
Common pathological mechanisms in neurodegenerative disease models. Alzheimer’s disease, Parkinson’s disease, Multiple Sclerosis, and Amyotrophic Lateral Sclerosis share key features, including chronic neuroinflammation, mitochondrial dysfunction, and oxidative stress. Image is adapted from Servier Medical Art https://smart.servier.com (accessed on 6 May 2026), licensed under CC BY 4.0 (https://creativecommons.org/licenses/by/4.0/, accessed on 6 May 2026).

In response to microenvironmental signals, microglia undergo both functional and morphological changes, adopting a reactive phenotype characterized by an amoeboid shape, enhanced motility, and increased release of pro-inflammatory mediators. While these responses are essential for host defense and tissue repair, their persistent activation represents a common hallmark of several neurodegenerative diseases (Figure 1). In this context, chronic neuroinflammation is increasingly recognized as a key driver of disease progression. In this review, four of the most common neurodegenerative diseases (AD, PD, MS and ALS) are taken into consideration, as they have been at the centre of many studies employing MSC-EVs.

- Alzheimer’s disease

AD is a progressive neurodegenerative disorder characterized by a gradual decline in cognitive and behavioural functions. Its main pathological hallmarks include extracellular accumulation of amyloid-β (Aβ) deposition and intracellular neurofibrillary tangles composed of hyperphosphorylated tau protein. These events ultimately result in synaptic dysfunction and neuronal loss [25,26]. AD represents a paradigmatic example of the interplay between protein aggregation and chronic neuroinflammation.

Beyond these classical pathological features, chronic neuroinflammation plays a dynamic role during disease progression. In early stages, inflammatory responses may exert a neuroprotective effect by promoting Aβ clearance and limiting tau propagation. However, as the disease progresses, microglial cells progressively lose their phagocytic capacity, leading to a sustained release of pro-inflammatory cytokines. In parallel, reactive astrocytes acquire neurotoxic phenotypes, and peripheral immune cells infiltrate the brain, further amplifying the inflammatory milieu [27].

In this context, a key contributor to AD pathogenesis is the inflammasome, a multiprotein intracellular complex predominantly expressed in myeloid cells that, together with the adaptor protein ASC, mediates the activation of the pro-inflammatory cascades. Fibrillar Aβ can directly activate NLRP3, promoting cytokine release and establishing a feed-forward inflammatory loop that exacerbates disease progression. Moreover, NLRP3 activation contributes to tau pathology and facilitates the spread of hyperphosphorylated tau [28].

Mitochondrial dysfunction further contributes to AD pathogenesis. Given the high energetic demands of neurons, mitochondrial homeostasis is essential for synaptic function and cell survival. Hyperphosphorylated tau, as well as Aβ plaques, disrupts mitochondrial dynamics and axonal transport, leading to synaptic failure, neuronal damage and ATP depletion [29]. Furthermore, mitophagy plays a key role in neuronal survival by maintaining healthy mitochondrial function and preventing neuronal damage. Although the exact role of mitophagy in the progression of AD is still unclear, proper mitophagy is known to be essential for maintaining neuronal homeostasis. Indeed, defective mitophagy leads to the accumulation of dysfunctional mitochondria, thereby promoting AD progression and memory loss. In turn, mitochondrial dysfunction can further amplify inflammatory signaling through the release of mitochondrial-derived danger signals, contributing to a self-perpetuating cycle of neurodegeneration [30].

Moreover, AD pathology is characterized by a profound dysregulation of the autophagy-lysosome pathway and endosomal trafficking, which are critical for the sequestration and degradation of neurotoxic Aβ and tau aggregates. Similarly to PD, the impairment of these clearance mechanisms leads to a “bottleneck” effect that facilitates protein accumulation and exacerbates cellular stress [25].

In this scenario, intercellular communication mechanisms, including EVs, are increasingly recognized as key mediators linking protein aggregation, mitochondrial dysfunction, and neuroinflammation in AD [31,32].

Collectively, these findings highlight neuroinflammation as a major therapeutic target in AD, with the potential to modulate disease progression by interfering with interconnected pathogenic pathways.

- Parkinson’s disease

PD is the second most common neurodegenerative disorder after AD and the most prevalent synucleinopathy. It is a chronic and progressive movement disorder characterized by motor symptoms, including resting tremor, rigidity, postural instability and bradykinesia. These clinical manifestations result from the progressive loss of dopaminergic neurons within the nigrostriatal pathway, and are accompanied by the accumulation of misfolded α-synuclein (α-syn) into Lewy bodies, which contribute to neurodegeneration [33]. In contrast to AD, PD is characterized by a marked selective vulnerability of dopaminergic neurons, which is closely linked to mitochondrial dysfunction and oxidative stress. Similarly to AD, PD is strongly associated with neuroinflammation, oxidative stress, and mitochondrial dysfunction, mainly within the Substantia Nigra *pars compacta* (SNpc), the region most affected by the pathology.

Activated microglia play a central role in PD pathogenesis by releasing neurotoxic factors and pro-inflammatory cytokines. These inflammatory signals have been detected in dopaminergic regions of PD patients. In addition, fibrillar α-synuclein can directly activate the NLRP3 inflammasome, promoting cytokine release and establishing a feed-forward loop that enhances α-synuclein aggregation and disease propagation [34].

As described for AD, also in PD, mitochondrial dysfunction contributes to neuroinflammation. The release of mitochondrial-derived danger signals, including mitochondrial DNA (mtDNA) and reactive oxygen species, can activate innate immune pathways and sustain microglial activation. Under physiological conditions, damaged mitochondria are selectively cleared through mitophagy to maintain cellular homeostasis. However, impairment of this process has been increasingly implicated in PD pathogenesis. In this context, EVs have been proposed as mediators of intercellular communication linking mitochondrial damage to inflammatory signaling, potentially through the transfer of mitochondrial components and stress-related signals [35,36,37].

Collectively, these mechanisms highlight the interplay between neuroinflammation and mitochondrial dysfunction in PD. Targeting these processes represents a promising therapeutic strategy to reduce inflammatory signaling, limit oxidative stress, restore mitophagy, and restrain the spread of α-synuclein pathology.

- Multiple Sclerosis

MS is the most common immune-mediated inflammatory disorder of the CNS. It is characterized by chronic neuroinflammation, axonal demyelination, and neurodegeneration. Unlike classical neurodegenerative diseases such as AD and PD, MS is primarily driven by immune-mediated mechanisms involving both central and peripheral immune components. Disease progression typically occurs through two main phases. An early stage is marked by persistent activation of microglia and astrocytes, leading to gradual neurological impairment. This phase is often followed by recurrent episodes of acute neurological dysfunction characteristic of relapsing-remitting MS, which may subsequently evolve into secondary progressive multiple sclerosis [38]. Persistent, low-grade inflammation within the CNS represents a major contributor to long-term disability [39].

Microglia and astrocytes play central roles in MS pathogenesis. Upon activation, microglia increase phagocytic activity and release of pro-inflammatory cytokines, contributing to synaptic dysfunction, demyelination and neurodegeneration. Activated astrocytes further promote gliosis and impair neuronal function. Importantly, a strong bidirectional crosstalk between microglia and astrocytes sustains and amplifies the chronic inflammatory environment observed in MS [40].

A distinctive feature of MS is the prominent involvement of peripheral immune cells. Disruption of BBB integrity allows infiltration of circulating lymphocytes and monocyte-derived macrophages into the CNS. These cells contribute to demyelination and axonal damage through antigen presentation and cytokine release. This interplay between resident glial cells and infiltrating immune populations represents a key driver of disease progression.

Inflammatory signaling pathways also play a critical role. Microglia are highly responsive to ROS and pro-inflammatory stimuli, which promote activation of the NLRP3 inflammasome and sustain chronic inflammation. Although inflammasome activation is a common feature across neurological disorders, in MS it is more closely linked to immune cell infiltration than to protein aggregation.

Mitochondrial dysfunction further contributes to disease progression. Impaired mitochondrial activity leads to increased ROS production, energy failure, and activation of apoptotic pathways. In both neurons and immune cells, mitochondrial alterations amplify inflammatory signaling, reinforcing the link between metabolic dysfunction and immune dysregulation [41].

Emerging evidence suggests that EVs may mediate communication between immune and neural cells by transferring inflammatory and mitochondrial signals, thereby influencing disease progression.

Collectively, these observations highlight the complexity of neuroinflammation and neurodegenerative disorders and underscore the need for therapeutic strategies capable of simultaneously targeting immune dysregulation, neuronal damage, mitochondrial damage and metabolic dysfunction.

- Amyotrophic Lateral Sclerosis

ALS is a neurodegenerative disease characterized by the progressive degeneration of upper and lower motor neurons (MNs), resulting in muscle atrophy, severe motor impairment, and death from respiratory failure within 2 to 5 years of diagnosis. ALS is a multifactorial condition influenced by both genetic and environmental factors; the vast majority of cases (90–95%) are sporadic, while approximately 10% are familial in origin [42,43].

The molecular mechanisms underlying ALS pathogenesis remain only partially understood. Key contributors include protein aggregation, dysregulation of RNA metabolism and splicing, metabolic alterations, and a prominent neuroinflammatory component.

Microglial and astroglial activation are hallmarks of ALS, alongside the infiltration of peripheral monocytes and lymphocytes. These processes typically precede or coincide with the onset of clinical symptoms and intensify as the disease progresses [44]. Microglial activation in ALS exists along a continuum between a neuroprotective (M2) and a neurotoxic (M1) phenotype (as discussed in Section 1.2). Moreover, multiple studies have reported reduced levels of CD4+ regulatory T cells (Tregs) in ALS patients, cells that are physiologically essential for modulating immune responses and maintaining immunological tolerance [45].

Oxidative stress and free radical production, arising from mitochondrial dysfunction, represent another critical dimension of ALS pathogenesis. Mitochondria are particularly important in MNs, which are among the largest cells in the body and bear exceptionally long axons. A range of mitochondrial alterations has been implicated in ALS pathophysiology, from oxidative stress to glutamate-mediated excitotoxicity. Together, these processes impair mitochondrial membrane potential, reduce ATP production, and drive neuronal degeneration from the earliest stages of the disease [46,47].

Taken together, these findings identify neuroinflammation and mitochondrial dysfunction as two major therapeutic targets in ALS, underscored by the central role of MNs in disease progression and their exceptionally high energy demands.

Given the shared pathological features across these neurodegenerative disorders, specifically chronic neuroinflammation and mitochondrial failure, MSC-derived Extracellular Vesicles (MSC-EVs) have emerged as a promising therapeutic strategy, offering several advantages over whole-cell approaches by preserving the intrinsic immunomodulatory and neuroprotective properties of their parent cells. Preclinical evidence has consistently shown that MSC-EVs can attenuate chronic neuroinflammation by suppressing pro-inflammatory cytokine secretion through a bioactive cargo that includes tetraspanins, receptors, integrins, and immunomodulatory miRNAs. Furthermore, an early preclinical study demonstrated that MSC-EVs can modulate pericyte responses by downregulating their neuroinflammatory secretome, including VEGFA, CXCL1, CXCL16, and TGF-β1—while upregulating mediators of vascular remodeling such as CXCL9, CXCL10, CD166, and IL-1ra [48].

Given that mitochondrial dysfunction is a primary driver of neuronal injury, particularly in the context of the high energetic demands of neurons, mitochondria-derived EVs (mito-EVs) represent an innovative complementary approach to restoring mitochondrial homeostasis. In support of this, Li et al. demonstrated that mito-EVs can be incorporated into damaged neuronal mitochondria and rescue inner mitochondrial membrane (IMM) function, thereby reducing both inflammation and oxidative stress [7,48].

For these reasons, a thorough characterization of the biological properties of MSCs, their derived EVs, and mito-EVs is essential to elucidate the mechanisms underpinning their therapeutic potential in CNS diseases [7].

## 2. MSC and MSC-Derived EVs: Characteristics and Therapeutic Potential

### 2.1. MSC and MSC-EVs

MSCs are multipotent stromal stem cells with broad therapeutic potential. They can be isolated from different tissues including umbilical cord, blood, Wharton’s jelly, placenta, skeletal muscle, dental pulp, skin and menstrual blood. Among these sources, bone marrow and adipose tissue are the most commonly used due to their accessibility and well-characterized biological properties [49]. Comprehensive reviews have extensively characterized MSC sources, differentiation potential, and their respective advantages and limitations [50].

MSCs can also derive from reprogrammed induced-pluripotent stem cells (iMSCs), potentially overcoming limited expansion capacity or donor-dependent variability. However, comparative studies report inconsistent results. Some describe functional similarity between iMSCs and primary MSCs, particularly those derived from umbilical cord [51], whereas others describe functional and genetic differences [52]. Despite this variability, iMSCs have demonstrated anti-inflammatory effects comparable to primary MSCs in experimental models of AD [53].

MSCs are widely recognized for their immunomodulatory and anti-inflammatory properties, making them attractive candidates for the treatment of neuroinflammatory and neurodegenerative disorders. However, direct MSC transplantation raises safety concerns, including potential tumorigenicity and immunogenicity. These limitations have driven the development of cell-free therapeutic approaches based on MSC-EVs. MSC-EVs maintain the beneficial properties of the parental cells, while minimizing risks associated with uncontrolled differentiation [54]. Owing to their lipid bilayer structure and low immunogenicity, MSC-EVs can cross the blood–brain barrier and reach the CNS. This makes them suitable for different delivery strategies, including non-invasive routes such as intranasal administration [55].

### 2.2. EV Biogenesis and Cargo

The therapeutic effects of MSC-EVs depend on both their biogenesis and their cargo. EVs comprise a heterogeneous population including Exos, MVs and apoptotic bodies, which differ in size, biogenesis pathways, and functional properties [14].

Exos are intraluminal vesicles (30–100 nm diameter) formed by inward budding of the endosomal membrane during multivesicular endosomes maturation and are released upon fusion with the membrane. MVs originate from outward budding of the membrane and range from 50 to 1000 nm, although tumor-derived forms can reach larger sizes and are often referred to as “oncosomes” [56]. Apoptotic bodies are released from dying cells through membrane blebbing. Interestingly, circulating apoptotic bodies have been implicated in MSC homeostasis and in the progression of several neurodegenerative pathologies such as AD, PD and Huntington’s disease [57].

MSC-EVs carry a broad spectrum of bioactive molecules, including microRNAs (miRNAs), proteins, lipids, metabolites, lncRNAs, mitochondrial components, and in some cases entire organelles. This molecular diversity underlies their ability to modulate inflammatory responses and synaptic function. In particular, MSC-EVs influence microglial and astrocytic activity, restore synaptic marker expression, and regulate complement system pathways involved in debris clearance and immune modulation [12].

The miRNA content of MSC-EVs appears to depend on the vesicle subtype. A study has identified at least three EV subpopulations based on membrane-associated ligands and cargo composition. Among them, ST-EVs (Shiga toxin B-binding EVs) are enriched in miR-191, miR-181, miR-22, miR-92, and miR-221. Notably, miR-221 is of interest due to its inhibitory effect on ADAM10, a protein involved in limiting amyloid-β accumulation [58,59]. Despite this, miR-221 has also been associated with broader roles in angiogenesis, inflammation, and apoptosis [60].

Beyond RNA cargo, MSC-EVs display distinct proteomic signatures depending on their tissue of origin, while retaining shared functional properties. All MSC-EVs are enriched in extracellular matrix-related and receptor-binding proteins. However, bone marrow-derived MSC-EVs are enriched in transport and migration-related proteins, adipose-derived MSC-EVs in metabolic and immune regulators, and umbilical cord-derived MSC-EVs in proteins involved in tissue regeneration, including collagen metabolism and cell adhesion [61,62]. These differences suggest that EV source selection may influence therapeutic outcomes depending on the target pathology. Proteomic analyses also distinguish Exos and MVs. Exos are enriched in proteins involved in extracellular matrix organization, integrin signaling, and immune responses, whereas MVs show higher abundance of mitochondrial, endoplasmic reticulum, and proteasomal proteins [63]. Lipidomic profiling further supports MSC-EV heterogeneity. Compared with oncosomes, MSC-EVs are enriched in cholesterol esters, phosphatidylserines, phosphatidylglycerols, phosphatidylinositols, acylcarnitines, cardiolipins, and lysophosphatidylcholines [63]. Although the functional role of most lipid species remains unclear, their composition likely contributes to membrane properties and EV functionality.

### 2.3. Mito-EVs as Emerging Mediators in Neuroinflammation and Neurodegeneration

Given the central role of mitochondrial dysfunction in neurodegeneration, a specialized EV subtype known as mito-EVs has recently been described [64]. These double-membraned EVs are enriched in mitochondrial proteins, electron transport chain components, pyruvate dehydrogenase complex subunits, and Krebs cycle enzymes. While these features suggest therapeutic potential for metabolic restoration, the biological impact of mito-EVs depends on their cellular origin. Cellular quality control mechanisms, including Parkin-mediated sorting, normally prevent the incorporation of damaged mitochondrial components into EVs, directing them toward lysosomal degradation [65]. However, this process may fail in pathological conditions, as seen in M1-polarized macrophages, allowing the export of dysfunctional mitochondria that promote oxidative stress and metabolic impairment in recipient cells [66].

Despite their therapeutic potential, mito-EVs isolation remains technically challenging due to low yields. To overcome this limitation, tissue-derived mitochondria-rich EVs (Ti-mitoEVs) have been developed from skeletal muscle, aiming to enhance mitochondrial content while reducing inflammatory damage, with potential applications in mitochondrial dysfunction-related diseases [67]. Alternatively, pharmacological approaches such as resveratrol administration may increase mitochondrial biogenesis and indirectly enhance mitochondrial content within EVs [68].

Among the different sources of mito-EVs currently under investigation, MSC-derived EVs are of particular interest for their capacity to transfer mtDNA and respiratory chain proteins (including ATP5A and ATP5B), facilitating the recovery of cellular bioenergetics in deficient cells [69]. This biological phenomenon reflects a broader role for MSCs in organelle trafficking and homeostatic support, often described as an “outsourcing” of mitophagy [10,70,71]. However, the extent to which entire, fully functional mitochondria are transferred via EVs, as opposed to fragments, remains a subject of ongoing investigation.

Finally, the EV landscape continues to expand with the discovery of rare populations like “blebbisomes” recently described by Jeppesen and colleagues [72]. These large vesicles (up to 20 µm) are naturally present in the bone marrow of healthy mice [72], contain mitochondria and multivesicular endosome-like compartments and have been proposed to participate in intercellular organelle exchange. Although still poorly characterized, they further illustrate the complexity of intercellular organelle trafficking during cellular stress and dysfunction [72].

### 2.4. Biological Functions of MSC-EVs and Mechanisms of Action in Neuroprotection and Neuroinflammation

Due to their nanoscale size and bioactive cargo, MSC-EVs efficiently cross the BBB and interact with resident neural and immune cells. This enables targeted modulation of pathological processes in diverse neurological disorders, including stroke, traumatic brain injury and neurodegeneration [53,73,74]. In experimental models of CNS injury, MSC-EVs exert consistent immunomodulatory and neuroprotective effects: they reduce glial activation and promote a shift toward anti-inflammatory phenotypes. This is accompanied by decreased levels of pro-inflammatory cytokines such as TNF-α and increased expression of anti-inflammatory mediators, including TGF-β and M2-associated markers [75,76,77,78,79]. At the molecular level, these effects are largely mediated by EV-associated bioactive cargo, including specific microRNAs (e.g., miR146a and miR124) and regulatory proteins. These molecules target key inflammatory pathways, including NF-κB and inflammasome signaling and PI3K/Akt. As a result, secondary neurotoxic cascades and apoptotic signaling are reduced [76,80]. In addition to proteins and nucleic acids, EV-associated lipids contribute to MSC-EV bioactivity. Lipid species such as sphingolipids, ceramides, and phospholipids actively regulate membrane dynamics and intercellular communication. They also influence inflammatory signaling within the CNS [56,81,82]. Interestingly, lipidomic analyses of microglia exposed to MSC-EVs revealed no major changes in the total lipid abundance. This suggests that EV effects are driven by qualitative rather than quantitative lipid remodeling. Mechanisms likely involve changes in membrane organization, lipid rafts, and bioactive lipid signaling, although these processes remain incompletely understood [82].

These immunomodulatory effects are accompanied by the suppression of neurotoxic A1 astrocyte activation and restoration of a more supportive glial environment. Together, these changes limit secondary injury cascades. In parallel, MSC-EVs promote neuronal survival, stimulate endogenous neural progenitor proliferation, and increase the expression of neurotrophic and angiogenic factors such as Brain-Derived Neurotrophic Factor (BDNF), Vascular Endothelial Growth Factor (VEGF), and Epidermal Growth Factor (EGF) [83]. Microglia remain central regulators of neuroinflammation in the CNS, alongside astrocytes and peripheral immune cells. They can adopt pro-inflammatory (M1-like) or anti-inflammatory (M2-like) phenotypes depending on environmental cues. Several studies have demonstrated that the administration of MSC-EVs can induce a switch from M1 to M2, attenuating neuroinflammation. This effect has been observed in LPS-stimulated microglia-like cells and in animal models of Alzheimer’s and Parkinson’s disease, with reduced pro-inflammatory cytokine release [55,84,85,86]. Mechanistically, this involves inhibition of inflammasome pathways, particularly NLRP3, leading to reduced pyroptosis and improved behavioral outcomes [87]. The miRNA miR-223-3p has been identified as a key regulator of this pathway, and its inhibition reverses several beneficial effects of MSC-EVs, including reduced amyloid deposition and neuronal apoptosis [53].

Astrocytes are also important mediators of neuroinflammation. They perform essential homeostatic functions, including metabolic support, BBB regulation, and debris clearance. Under pathological conditions, astrocytes shift toward a pro-inflammatory A1 phenotype. MSC-EVs can counteract this transition, reducing astrocytic activation and inflammatory signaling in neurodegenerative contexts [87,88]. Beyond glial modulation, MSC-EVs exert direct effects on endothelial and neuronal cells. EVs enriched in miR-146a reduce oxidative stress-induced endothelial senescence and improve angiogenic responses. This may also contribute to BBB stabilization [89]. Finally, MSC-EVs promote neuronal survival and functional recovery by reducing oxidative stress and restoring redox homeostasis through activation of cytoprotective pathways such as Nrf2/HO-1 [74,89]. In stroke models, intranasal administration reduces glial scar formation and improves motor outcomes [90]. In neonatal hypoxic–ischemic injury, MSC-EVs enhance oligodendrocyte maturation, myelination, and white matter integrity, supporting brain repair processes [83,91]. Consistently, MSC-EVs have been shown to enhance endogenous repair mechanisms, including neurite outgrowth, synaptic plasticity, oligodendrocyte maturation, and remyelination. Together, these effects contribute to functional recovery across multiple CNS injury models [92]. Emerging evidence also suggests that MSC-EVs modulate cellular metabolism and mitochondrial function in recipient cells [93]. Through transfer of mitochondrial-related proteins, mitochondrial DNA, and metabolic regulators, EVs may restore balance and reduce oxidative stress [94,95]. This is particularly relevant in neurodegenerative diseases, where mitochondrial dysfunction represents a key pathological hallmark. In this context, mito-EVs may represent an additional mechanism of intercellular metabolic communication.

### 2.5. Limitations of “Naïve” MSC-EVs

Although MSC-EVs show considerable promise as a cell-free therapeutic approach for neuroinflammatory and neurodegenerative disorders, naïve, unmodified MSC-EVs face several critical limitations that may hinder their translational success [96]. First, MSC-EV populations are inherently heterogeneous, with considerable variability in size, cargo composition, and functional potency across batches; this variability, heavily influenced by tissue sources and isolation methods, represents a major obstacle to reproducibility and standardization [97,98]. Second, naïve MSC-EVs often exhibit limited targeting specificity to key CNS cell types involved in neurodegeneration, as their uptake remains largely non-specific in the absence of surface engineering, thereby reducing their ability to modulate pathological processes in disease-relevant brain regions [99]. Finally, naïve MSC-EVs display suboptimal stability and retention in vivo because they are rapidly cleared by peripheral organs following systemic administration, resulting in a short circulation half-life and reduced CNS bioavailability. This limits their therapeutic efficacy in neurodegenerative settings characterized by chronic and progressive pathology. Together, these limitations highlight the need for optimization strategies, including cargo engineering, surface modification, and parental cell preconditioning to improve consistency, targeting efficiency, and in vivo stability of MSC-EVs. To overcome these challenges, several bioengineering approaches have been developed. Preconditioning of parental MSCs under hypoxic conditions enhances neuroprotective EV cargo, leading to improved synaptic preservation, cognitive performance, and reduced amyloid-β accumulation in AD models [100]. Another strategy involves therapeutic cargo loading, in which MSCs are genetically engineered to overexpress specific miRNAs (e.g., miR-29b, miR-22). Their enrichment in EVs promotes the downregulation of BACE1 and other AD-related targets, improving cognitive outcomes and attenuating disease-associated signaling [98,99]. In parallel, surface functionalization strategies, including the incorporation of targeting peptides or ligands, can enhance EV uptake by neurons and glial cells, thereby improving delivery efficiency to CNS-relevant cell populations and addressing the limited specificity of naïve EVs [100]. Finally, alternative administration routes, such as intranasal delivery, have been explored to increase CNS accumulation while bypassing systemic clearance mechanisms, leading to reduced amyloid plaque burden and improved cognitive performance in 5xFAD mice [55]. Collectively, these engineering approaches improve MSC-EVs stability, targeting specificity, and therapeutic cargo delivery, making engineered MSC-EVs a more promising strategy for treating multifactorial neurodegenerative pathologies compared with naïve EVs.

## 3. Preconditioning Strategies to Enhance MSC-EV Therapeutic Actions

### 3.1. Hypoxic/Anoxic Preconditioning

Although MSC-EVs exert intrinsic anti-inflammatory and neuroprotective effects, several preconditioning strategies have been explored in recent years to further enhance their therapeutic potential, including hypoxic/anoxic exposure and cytokine priming.

A common feature across different preconditioning strategies is the ability of MSC-EVs to modulate innate immune responses, particularly by promoting the shift in microglia and macrophages from a pro-inflammatory toward an anti-inflammatory state, which represents a key mechanism underlying their therapeutic effects.

Hypoxic preconditioning has emerged as one of the most effective approaches to potentiate MSC-EV functions [101]. Importantly, hypoxia-primed EVs (hyMSC-EVs) retain a favorable safety profile, as exposure to low oxygen conditions does not appear to compromise their tolerability [102]. As mentioned above (Section 2.2), hyMSC-EVs maintain the ability to modulate microglial activation, promoting an anti-inflammatory phenotype. This effect has been demonstrated both in vitro and in vivo, including in spinal cord injury (SCI) models following systemic administration. Interestingly, this immunomodulatory effect has been associated with the enrichment of specific miRNAs, particularly miR-146-5p, suggesting that this molecule might play a role in macrophage and microglial polarization [87]. Consistent findings have been reported in animal models of experimental autoimmune encephalomyelitis (EAE), where miR-146a-enriched hyMSC-EVs were associated with a significant attenuation of neuroinflammation, decreased pro-inflammatory cytokines (e.g., TNF-α, IFN-γ) and increased expression of anti-inflammatory mediators such as IL-10. Additional miRNAs, including miR-145-5p and miR-216a-5p, have also been implicated in the neuroprotective and anti-inflammatory effects induced by hypoxic preconditioning [103]. Overall, these findings suggest that hypoxic preconditioning enhances both EV production and cargo composition, thereby strengthening the anti-inflammatory potential of MSC-EVs. However, it should be noted that most of these observations derive from preclinical models, and their translatability across different neurodegenerative conditions remains to be fully established.

Similar effects have also been observed in astrocytes. In rat models of SCI, administration of hyMSC-EVs promoted the transition from A1 to A2 neuroprotective phenotype, enhancing motor recovery and the anti-apoptotic/anti-inflammatory responses compared to control MSC-EVs [104]. Taken together, this evidence indicates that hypoxic preconditioning enables MSC-EVs to target multiple components of neuroinflammation, including both microglia and astrocytes, thereby making it a feasible strategy to boost the potential for MSC-EVs to address neuroinflammatory diseases.

Beyond their immunomodulatory effects, hypoxia-primed MSC-EVs also exhibit enhanced pro-angiogenic properties. This effect is mediated by coordinated changes in both miRNA cargo and protein signaling pathways. In particular, hyMSC-EVs modulate endothelial cell function by altering the expression of angiogenesis-related miRNAs, including the upregulation of miR-126-3p and miR-140-5p, alongside the downregulation of miR-186-5p, miR-370-3p and miR-409-3p. These changes are associated with distinct transcriptomic profiles in recipient endothelial cells, particularly involving extracellular matrix-receptor interactions and focal adhesion pathways, which collectively contribute to enhanced vascular remodeling and tissue repair [105]. Beyond miRNA-mediated regulation, hypoxic preconditioning also activates key pro-angiogenic signaling pathways. Notably, the upregulation of HIF-1α, a central regulator of cellular responses to hypoxia, promotes the expression of VEGF, thereby stimulating angiogenesis [106]. HIF-1α not only plays a central role in angiogenesis but also interacts with survival pathways such as Akt, further supporting cell survival and tissue regeneration [107].

Overall, these findings suggest that hypoxic preconditioning enhances the pro-angiogenic capacity of MSC-EVs through a combination of miRNA remodeling and activation of hypoxia-responsive signaling pathways, ultimately contributing to improved neurovascular function and recovery. At the cellular level, hypoxia-primed MSC-EVs also contribute to the regulation of mitochondrial function. In particular, hyMSC-EVs have been shown to attenuate mitochondrial dysfunction by reducing oxidative stress and promoting mitophagy, thereby supporting cellular homeostasis. These effects are partly mediated by the enrichment of specific proteins and miRNAs within EV cargo. For instance, hyMSC-EVs are enriched in Parkinson’s disease protein 7 (PARK7/DJ-1), a key antioxidant agent that reduces mitochondrial ROS and protects against oxidative damage [108]. This is particularly relevant in the context of neurodegenerative diseases, where mutations in PARK7 can be associated with familial forms of PD. In parallel, hypoxic preconditioning increases the levels of miR-214-3p, which has been implicated in the regulation of the PI3K/AKT/mTOR pathway. Through this mechanism, miR-214-3p contributes to the maintenance of mitochondrial stability and promotes mitophagy, facilitating the removal of damaged mitochondria [109].

Collectively, these findings suggest that hyMSC-EVs may exert neuroprotective effects not only through immunomodulation but also by directly targeting mitochondrial dysfunction, a key pathological feature of many neurodegenerative disorders.

### 3.2. Inflammatory Preconditioning

Inflammatory preconditioning represents a complementary strategy to hypoxia, based on the exposure of MSCs to pro-inflammatory cytokines such as tumor necrosis factor-α (TNF-α), interferon-ϒ (IFN-γ) and interleukin-1β (IL-1β). This approach aims to mimic the pathological microenvironment, thereby enhancing the immunomodulatory and anti-inflammatory properties of MSC-EVs. Interestingly, several studies have shown that cytokine priming can increase both EV production and functional potency. However, the magnitude and nature of these effects appear to be highly dependent on the specific combination of cytokines, exposure conditions, and MSC source, contributing to variability across studies.

Among the different stimuli, TNF-α has been extensively investigated and has been shown to enhance EV release while promoting macrophage polarization toward an anti-inflammatory state, partly through increased CD73 expression [85]. Interestingly, comparative studies indicate that different preconditioning strategies can exert distinct effects on the MSC secretome. For example, inflammatory priming has been reported to suppress the pro-angiogenic activity of soluble factors, while largely preserving the angiogenic potential of EVs. In contrast, hypoxic preconditioning appears to enhance pro-angiogenic responses, highlighting functional divergence between these approaches [110]. Overall, these observations suggest that the choice of preconditioning strategy may need to be tailored to the desired therapeutic outcome, depending on whether immunomodulation, angiogenesis, or other regenerative processes are the primary target.

For instance, TNF-α preconditioning has been shown to enhance autophagy-related pathways in MSC-EVs, further contributing to their cytoprotective effects. In particular, MSC-EVs derived from TNF-α-stimulated MSCs can increase the expression of ATG16L1, a key regulator of autophagosome formation, suggesting an activation of autophagic flux in recipient cells. This effect appears to be at least partially mediated by the PI3K/Akt signaling pathway, as it is significantly attenuated when LY294002, a PI3K inhibitor, is co-administered with these EVs. Interestingly, this observation contrasts with findings from hypoxic preconditioning, where modulation of the PI3K/Akt/mTOR axis is often associated with inhibition rather than activation of downstream signaling. These discrepancies highlight how distinct priming strategies may differentially regulate autophagy and survival pathways, depending on the cellular context and molecular targets involved [109,111].

On the other hand, some effects appear to be conserved across different priming strategies. Indeed, both hypoxic and TNF-α preconditioning have been consistently associated with the upregulation of miR-146a, a key anti-inflammatory miRNA often implicated across different priming strategies. These findings suggest common regulatory mechanisms, although their functional relevance remains unclear [112,113].

Similarly, IL-1β preconditioning has been shown to reproduce part of the anti-inflammatory effects observed with other cytokine-based priming strategies. In particular, it can induce an upregulation of miR-146a, alongside a shift in macrophage polarization toward the M2 phenotype. Functional studies further support a role for miR-146a in this process, as pharmacological inhibition of this miRNA partially reverses the M1-to-M2 transition, suggesting its contribution to cytokine-driven immunomodulation. Overall, these findings support the idea that distinct inflammatory stimuli may converge on a limited number of key regulatory miRNAs, such as miR-146a, which act as central mediators of the anti-inflammatory effects of primed MSC-EVs [114].

Interestingly, another miRNA that is supposed to mediate the anti-inflammatory and neuroprotective effects of TNF-α-primed MSC-EVs is miR-21a-5p, whose production is boosted by this preconditioning technique. This molecule not only reduces the pro-inflammatory cytokine expression, but it can also interact downstream with the programmed cell death 4 molecule (PDGD4), suppressing its expression and preventing apoptosis since PDGD4 is an inhibitor of the PI3K/AKT/GSK-3β signaling pathway [115].

IFN-γ represents another relevant cytokine for MSC priming. MSC-EVs preconditioned with IFN-γ display enhanced anti-inflammatory properties compared to naïve EVs and have shown therapeutic potential in the treatment of EAE, where they improve functional outcomes in murine models. These effects are accompanied by reduced demyelination and induction of immune tolerance by an increased proportion of regulatory T cells alongside a reduction in cytotoxic T cells, findings that are particularly relevant in the context of MS [116]. Consistently the use of IFN-γ-primed MSC-EVs also reduces neuroinflammatory markers in the spinal cord of EAE mice and partially restores the phenotype of activated microglia in an Amyotrophic Lateral Sclerosis (ALS) model, including the downregulation of TNF-α and IL-1β [117].

A synergistic strategy has been proposed by combining IFN-γ with TNF-α during MSC preconditioning [55]. This combined stimulation enhances the anti-inflammatory profile of MSC-EVs both in vitro and ex vivo, and modulates their miRNA cargo without significantly altering their lipid composition. In particular, EVs derived from dual-primed MSCs show upregulation of several immunoregulatory miRNAs, including miR-155-5p, miR-324-5p, miR-92b-3p, and miR-146a-5p, reinforcing the idea that inflammatory priming can reshape EV-associated regulatory networks toward an anti-inflammatory phenotype. Interestingly, miR-221-3p is also increased despite its reported detrimental role in AD, highlighting the context-dependent nature of miRNA function across different pathological settings.

Nevertheless, this combined primary strategy also enhances the antioxidant and anti-apoptotic properties of MSC-EVs, as reflected by increased NQO1 expression and reduced cleaved caspase-3 levels [118,119]. Collectively, these findings suggest that multi-cytokine priming may represent a more effective approach than single-factor stimulation, as it enables simultaneous modulation of inflammatory, oxidative, and survival pathways, thereby broadening the therapeutic potential of MSC-EVs.

However, it should be noted that, although several studies report changes in miRNA expression following priming, others have reached contrasting conclusions, suggesting that these strategies may not substantially alter the overall miRNA landscape of MSC-EVs, but rather affect a limited number of specific miRNAs [120].

More broadly, the current literature is limited by a lack of standardized experimental conditions and direct comparative studies across different priming strategies, which complicates the identification of robust and reproducible molecular signatures. Taken together these limitations highlight that, despite promising functional effects, the mechanistic basis of MSC-EV priming remains incompletely defined and requires further systematic investigation.

### 3.3. Other Preconditioning Strategies

Beyond hypoxic and cytokine-based priming, additional strategies have been proposed to further modulate MSC-EV composition and function. These approaches include pharmacological and genetic interventions, although their mechanistic characterization and comparative validation remain more limited.

#### 3.3.1. Drugs and Nutraceuticals

Among pharmacological modulators, melatonin has gained considerable attention in MSC priming. Melatonin, a circadian rhythm-related hormone, enhances the immunomodulatory capacity of MSC-EVs by promoting macrophage polarization toward an anti-inflammatory M2 phenotype via activation of the PTEN/AKT pathway. This results in reduced secretion of pro-inflammatory cytokines, including TNF-α and IL-1β, and increased expression of IL-10 [103]. Moreover, melatonin-preconditioned MSC-EVs have been reported to promote angiogenesis and exert antioxidant and anti-apoptotic effects, partly recapitulating observations from hypoxic and cytokine-based priming strategies. These effects have also been associated with improved functional recovery in experimental models of SCI [121,122], suggesting partial overlap in downstream biological outcomes across different priming approaches, despite distinct upstream mechanisms.

Other pharmacological agents such as curcumin and metformin have also been explored. Curcumin-preconditioned MSC-EVs have been shown to improve both memory and spatial learning deficits in rat models of AD, likely through the reduction in neuroinflammation and the promotion of microglial polarization toward the M2 phenotype [123]. However, the extent to which these effects depend on EV cargo remodeling versus indirect modulation of donor MSC state remains incompletely defined. Metformin enhances MSC-EV secretion through autophagy-related mechanisms, including amphisome formation, a process involving fusion between autophagosomes and multivesicular bodies, thereby influencing EV biogenesis and release. In addition, metformin-preconditioned MSC-EVs have been associated with modulation of senescence-associated markers and cell growth-related pathways, as shown by proteomic and Gene Ontology analyses [124], suggesting that pharmacological priming may act at both the secretory and functional level of MSC biology.

#### 3.3.2. Genetic Engineering

A final strategy to enhance the therapeutic potential of MSC-EVs is genetic engineering, which exploits EVs as customizable nanocarriers to target specific pathological pathways. SHP2-enriched MSC-EVs have been shown to modulate apoptosis, NLRP3 inflammasome activation, and mitophagy, resulting in improved synaptic integrity and cognitive performance in an AD model. Treated mice showed behavioral improvements comparable to wild-type animals in tests such as the Morris Water Maze and open field paradigm, indicating substantial functional rescue [125]. Similarly, loading MSC-EVs with a miR-206-3p antagomir has been reported to increase BDNF expression and improve cognitive outcomes, supporting enhanced neurogenesis and synaptic plasticity [126].

Beyond cargo modification, genetic engineering can also be used to increase EV yield. Kojima and colleagues identified three genes (STEAP3, SDC4, and NadB) that promote exosome biogenesis and developed a tricistronic plasmid (Exosome Production Booster), which significantly enhances exosome production and release across multiple cell lines, including human MSCs [127]. This strategy addresses one of the major translational limitations of EV-based therapies, namely scalability, although its applicability to clinical-grade production still requires validation.

Finally, EV loading strategies have been exploited to target specific inflammatory pathways. For instance, delivery of miR-223 into MSC-EVs via electroporation has been shown to suppress NLRP3 inflammasome activation, reducing inflammatory signaling and cell death [128]. However, the efficiency, stability, and clinical feasibility of such loading approaches remain areas that require further standardization.

## 4. Enhanced Cargo in Preconditioned MSC-EVs and Mito-EVs

The use of MSC-EVs represents a promising strategy for treating chronic neuroinflammation in various neurodegenerative diseases and regenerative medicine applications. EVs act as carriers of bioactive molecules, including lipids, proteins, and specific microRNAs capable of modulating immune cell infiltration and promoting macrophage polarization toward anti-inflammatory phenotypes [129]. Preconditioning strategies can further enhance these effects by reshaping EV cargo and increasing regulatory mediators involved in inflammation control, cell survival, and microglial polarization toward an M2-like state [130,131]. In this context, mito-EVs represent a distinct and emerging class of EVs capable of transferring mitochondrial components including mtDNA, thereby contributing to restoration of bioenergetic balance, ATP production, and reduction in oxidative stress [67,132]. However, their functional role is context-dependent, as mito-EVs may also reflect or propagate mitochondrial dysfunction depending on the metabolic state of the donor cell.

### 4.1. Protein Cargo Remodeling MSC-EVs and Mito-EVs

MSC-EVs express conserved surface markers, including CD73, CD90 and CD105, together with structural and adhesion-related proteins such as vimentin, actinin-1, and type I–VI collagens, which facilitate EV uptake and intercellular communication [62]. Preconditioning with inflammatory or environmental stimuli significantly changes protein cargo, enhancing their immunomodulatory and neuroprotective properties.

In particular, TNF-α priming increases anti-inflammatory mediators such as IL-10 and arginase-1, promoting macrophage polarization toward an M2 phenotype [133]. Stress-related proteins such as HSP-70 are also modulated by preconditioning and contribute to protein homeostasis and neuroprotective responses [133].

Consistently, MSC-EVs exert neuroprotective effects partly through TNF-α downregulation and enhanced delivery of neurotrophic factors such as BDNF, VEGF, NGF, and EGF [83]. However, the mechanisms governing selective growth factor enrichment remain poorly characterized.

**Figure 2:**
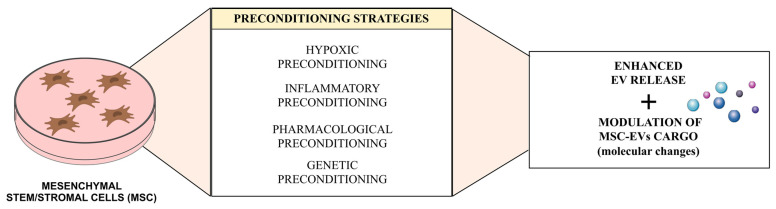
Effects of preconditioning strategies on MSC-derived EVs. Preconditioning can enhance EV release and modulate MSC-EV cargo through molecular changes. Image is adapted from Servier Medical Art https://smart.servier.com (accessed on 6 May 2026), licensed under CC BY 4.0 (https://creativecommons.org/licenses/by/4.0/, accessed on 6 May 2026).

Only a limited number of studies have directly addressed strategies to enhance neurotrophic cargo in MSC-EVs. Among these, the work by Kim et al. reported peptide-loaded MSC-EVs that increase BDNF expression and promote neurogenesis under hypoxic conditions [134], while Lian et al. described NGF-preconditioned EVs integrated into 3D scaffolds, improving axonal growth and Schwann cell function (Figure 2) [135].

Within this context, mito-EVs represent a specialized subpopulation containing mitochondrial components such as voltage-dependent anion channel (VDAC), cytochrome-C oxidase-IV (COX-IV), and pyruvate dehydrogenase-E1α (PDH-E1α) [64]. By transferring mtDNA and electron transport chain proteins, they support metabolic recovery, reduce oxidative stress, and improve proteostasis, thereby counteracting amyloid-β and tau pathology [67]. However, mito-EVs may also reflect the metabolic state of donor cells. In pathological conditions such as AD, they can become enriched in monoamine oxidase-B (MAO-B), contributing to oxidative stress, monoamine degradation and synaptic dysfunction. Thus, mito-EVs may act as both mediators of homeostasis and dysfunction depending on their composition [64].

Overall, preconditioning reshapes MSC-EV protein cargo, enhancing neuroprotective functions, while mito-EVs provide a functional link between mitochondrial status and neuronal fate.

### 4.2. miRNA Modulation and Emerging Role of lncRNAs

While protein cargo remodeling contributes substantially to the functional effects of MSC-EVs, growing evidence indicates that RNA components, particularly miRNAs, represent an additional and highly dynamic layer of regulation.

The neuroprotective and immunomodulatory effects of MSC-EVs are primarily mediated by their RNA cargo, particularly small non-coding RNAs. Among these, miRNAs play a central role in regulating gene expression in recipient cells, thereby modulating neuroinflammation, neuronal survival and autophagy.

The selective enrichment of specific miRNAs into EVs is regulated by RNA-binding proteins that control RNA sorting and export. However, EV-associated RNA populations are heterogeneous and include not only miRNAs but also tRNA fragments, Y-RNAs, snRNAs, and rRNA-derived species, which may represent a substantial fraction of EV cargo [136].

In neuroinflammatory conditions, MSC-EVs exert protective effects through miRNA-mediated modulation of inflammatory signaling (see Section 2.2). In ALS models, MSC-EVs reduce astrocyte-mediated neurotoxicity and oxidative stress by inhibiting the NLRP3 inflammasome, with effects mediated by miR-466q, miR-467f, and miR-29b-3p through regulation of the p38 MAPK pathway [126]. EV production and functional activity can be further enhanced by priming strategies, including IFN-γ stimulation and ATP supplementation [137].

Among EV-associated miRNAs, miR-21a-5p emerges as a key regulator of immunomodulation. It promotes a shift toward an anti-inflammatory microglial phenotype and suppresses STAT3 signaling, resulting in reduced expression of pro-inflammatory cytokines such as TNF-α and IL-1β [138].

However, several strategies can be employed to enhance the therapeutic potential of MSC-EVs, including approaches aimed at the engineering of EVs. It has been reported that miR-124 delivered by MSC-EVs exerts neuroprotective effects by stabilizing the p62/Keap1/Nrf2 signaling axis and restoring autophagic balance, thereby reducing oxidative stress [139]. Similarly, the delivery of miR-132-3p enhances synaptic function and neuroprotection through activation of the Ras/Akt/GSK-3β pathway via RASA1 inhibition, leading to reduced amyloid-β accumulation and tau pathology [140]. miR-146a-5p has also been identified as a key mediator of neuronal functional recovery. Notably, MSC-EVs engineered–enriched in miR-146a-5p exhibit enhanced anti-inflammatory efficacy compared to naïve MSC-EVs. This effect is primarily linked to the ability of miR-146a-5p to directly target and downregulate TNF Receptor-Associated Factor 6 (TRAF6) and Interleukin-1 Receptor-Associated Kinase 1 (IRAK1) and suppress NF-κB signaling [141].

Beyond miRNAs, long non-coding RNAs (lncRNAs) are increasingly recognized as functional components of MSC-EVs. These transcripts can regulate gene expression in recipient cells through competing endogenous RNA (ceRNA) mechanisms, influencing inflammation, cell survival, and regeneration. For example, lncRNA H19 promotes neuroregeneration and reduces oxidative stress via activation of Wnt/β-catenin signaling, while lncRNAs such as *MALAT1* and *NEAT1* have been implicated in modulating EV-mediated anti-inflammatory responses [142].

Collectively, MSC-EVs act as RNA delivery systems capable of coordinating complex regulatory networks in target cells. However, the relative contribution of different RNA species and the mechanisms governing their selective packaging remain incompletely understood. Greater standardization and comparative studies will be essential to define robust RNA signatures associated with therapeutic efficacy [117].

## 5. Bioavailability and MSC-EV Administration Routes

Bioavailability remains a major challenge for therapeutic applications of MSC-EVs. After systemic administration, MSC-EVs display a short circulation half-life and are rapidly cleared by the mononuclear phagocyte system, leading to predominant accumulation in peripheral organs such as the liver, spleen and lungs, with only a limited fraction reaching the CNS [143].

Early systematic in vivo studies by Wiklander and colleagues [144] demonstrated that EV biodistribution is strongly influenced by their cellular origin, indicating that bioavailability is not uniform but depends on EV source. In addition, the route of administration critically affects EV circulation time and tissue exposure, with intravenous, intraperitoneal, and subcutaneous delivery leading to distinct distribution profiles. Despite detectable systemic distribution, EV accumulation remains largely confined to clearance-associated organs. Notably, EVs were not immediately degraded after administration, remaining detectable at early time points and indicating short-term stability in vivo.

These biodistribution patterns represent a major limitation for CNS targeting. Although MSC-EVs are capable of crossing the BBB, likely via transcytosis and receptor-mediated mechanisms, this process is relatively inefficient under physiological conditions [145,146,147]. BBB dysfunction in neurodegenerative diseases may partially facilitate EV entry, but does not fully compensate for rapid systemic clearance.

To overcome these limitations, alternative delivery strategies have been explored. Intranasal administration has emerged as a promising approach, enabling direct nose-to-brain transport while bypassing the BBB. Preclinical studies have shown that this route enhances cerebral EV accumulation and is associated with reduced amyloid-β burden and improved cognitive performance in AD models [55]. In contrast, intracerebral or intrathecal delivery ensures high local EV concentrations, but remains invasive and clinically less feasible [143]. Notably, intracerebral administration has been shown to significantly reduce amyloid plaque burden in early-stage AD models [78,148], highlighting the trade-off between efficacy and translational applicability.

Further insight into EV brain delivery was provided by Banks et al. [146], who demonstrated that peripherally administered EVs can cross the BBB via active, saturable transport mechanisms. Importantly, EV uptake into the brain was dynamically modulated by inflammatory states, with systemic inflammation significantly enhancing BBB transport. Together with the findings of Wiklander et al. [144], these findings indicate that EV brain availability is a regulated and context-dependent process rather than a passive phenomenon.

Despite this evidence, important limitations remain. EV biodistribution is still largely dominated by uptake in peripheral clearance-associated organs, and baseline EV brain availability may be insufficient for therapeutic efficacy. Furthermore, inflammation-dependent variability further complicates predictability of EV delivery across pathological conditions. Methodological issues, including the use of labeled EVs, introduce potential artifacts affecting biodistribution analysis.

Collectively, these observations indicate that while MSC-EVs are bioavailable in vivo, achieving effective and tissue-specific delivery to the brain remains a major bottleneck. Future efforts should focus on improving targeting efficiency, minimizing off-target clearance, optimizing delivery routes, and standardizing experimental approaches to better define pharmacokinetics and therapeutic dosing.

## 6. Therapeutic Applications Across Disorders

The therapeutic efficacy of EVs derived from in vitro MSC cultures has been well-established, with these vesicles reproducing the beneficial effects of their parent MSCs in numerous in vitro functional assays and pre-clinical models of disease [149].

Importantly, the therapeutic reproducibility of MSC-EVs relies on standardized approaches for their isolation and characterization. Historically, differences in EV nomenclature, isolation procedures, and characterization criteria have represented major barriers to clinical translation. To address these challenges, consensus guidelines have been developed to define the key parameters for MSC-derived EVs intended for therapeutic use, thereby facilitating quality control, comparability among studies, and regulatory approval [150]. In this context, MSC-EVs have demonstrated considerable therapeutic potential in preclinical models of neuroinflammation and neurodegenerative diseases, as discussed in the following paragraphs.

### 6.1. Alzheimer’s Disease

The therapeutic relevance of MSC-EVs was initially supported by clinical evidence demonstrating that MSCs can exert potent immunomodulatory and tissue-protective effects in otherwise treatment-refractory conditions, such as acute graft-versus-host disease [151]. These observations prompted extensive investigation into MSC-EVs as a cell-free therapeutic modality, leading to a growing body of preclinical studies exploring their efficacy in neurodegenerative disorders, including AD. Collectively, MSC-EVs recapitulate many of the beneficial effects of parental MSCs, while offering advantages in terms of safety, scalability and tissue penetration.

In experimental models of AD, MSC-EVs act by targeting multiple disease-relevant pathways. Early in vitro studies showed that adipose-derived MSC-EVs reduce neuronal apoptosis and attenuate Aβ pathology in neuronal cultures from transgenic AD mice, supporting a direct neuroprotective effect [152]. Mechanistically, MSC-EVs can contribute to Aβ clearance through the delivery of proteolytic enzymes such as neprilysin, reducing both extracellular and intracellular Aβ levels in APP-expressing neurons [78,153]. Intracerebral or systemic administration of MSC-EVs reduces Aβ plaque burden, synaptic loss, and dystrophic neurites in transgenic AD mouse models such as APP/PS1 and 3xTg mice, accompanied by improvements in cognitive performance [15,55,78,143,154,155]. These effects are consistently associated with attenuation of neuroinflammation, including reduced microglial and astrocytic activation and decreased pro-inflammatory cytokine levels. In parallel, MSC-EVs support synaptic preservation, as reflected by restoration of synaptophysin and PSD-95 expression and improved dendritic architecture [15,155,156,157].

Beyond amyloid and inflammatory pathways, emerging evidence points to an additional role of MSC-EVs in regulating neuronal bioenergetics. In particular, increasing attention has focused on specialized EV subpopulations, including mito-EVs that have been linked to improved mitochondrial function, reduced oxidative stress and restoration of energy metabolism in AD models [15,155,157]. Consistently, neural stem cell-derived EVs (NSC-EVs) have been shown to improve cognitive performance in APP/PS1 mice while restoring mitochondrial function and synaptic integrity. NSC-EV treatment enhanced mitochondrial biogenesis and stress-response pathways (PGC-1α, NRF1/2, SIRT1), increased synaptic protein expression, and reduced oxidative stress, neuroinflammation, and microglial activation. Notably, these beneficial effects occurred independently of changes in amyloid-β burden, highlighting a bioenergetic and synaptic mechanism of action [158].

Although direct evidence for mito-EV efficacy in AD remains limited, alterations in mitochondrial EV signatures have been reported in neurodegenerative diseases, suggesting a role for EV-mediated mitochondrial signaling in disease progression or compensation. Within this framework, mitochondrial cargo modulation may represent an additional layer of MSC-EV therapeutic activity, complementing their established anti-amyloid and anti-inflammatory effects.

Taken together, these findings support a multifaceted model of MSC-EV-mediated neuroprotection in AD, encompassing coordinated effects on amyloid processing, neuroinflammation, synaptic integrity and cellular metabolism. Integration of mitochondrial biology into MSC-EV-based therapeutic strategies may therefore represent a promising direction for future therapeutic development in AD and related neurodegenerative disorders.

### 6.2. Parkinson’s Disease

MSC-EVs have been proposed as a therapeutic strategy for PD due to their protective properties, which translate into reduced microglial activation, improved motor behavior and decreased α-syn accumulation, ultimately supporting nigro-striatal pathway integrity. Moreover, MSC-EVs promote autophagy, as indicated by the decreased p62 and increased LC3-II levels [86,159].

Building on these baseline effects, several strategies have been developed to enhance MSC-EV efficacy also in PD. One approach involves the combination of UC-MSC-EVs with nanoliposomes loaded with baicalein and oleuropein, two inhibitors of α-syn fibrillation. This hybrid system significantly reduces α-syn aggregation, decreases oxidative stress, promotes cell viability in vitro and improves intracellular delivery efficiency compared with nanoliposomes alone, while also exerting a beneficial effect on angiogenesis [160].

Similarly, curcumin-loaded MSC-EVs (PR-EXO/PP@Cur), delivered via intranasal administration, have been shown to reduce α-syn accumulation and neuroinflammation, leading to functional recovery at both neuronal and behavioral levels [126]. However, the exact contribution of EV-mediated versus cargo-driven effects remains to be fully clarified.

Other bioactive compounds have also been incorporated into MSC-EVs. For instance, dihydrotanshinone-loaded CCr2-enriched MSC-EVs improved motor performance and reduced neuroinflammation in a 1-methyl-4-phenyl-1,2,3,6-tetrahydropyridine (MPTP)-induced PD model. This approach limited peripheral immune cell infiltration. Mechanistically, this effect involves NRF2 activation and partial suppression of ferroptosis in microglia, a relevant mechanism in the context of iron accumulation in PD brains [161].

Neurotrophic factor engineering has also been explored. BDNF-loaded MSC-EVs improve motor deficits and reduce dopaminergic neuron loss in 6-OHDA models, also engaging NRF2 signaling and contributing to ferroptosis inhibition, similarly to other antioxidant-based strategies [162].

Finally, more advanced engineering approaches have been proposed to enhance targeting and intracellular delivery. Kojima et al. developed exosome-producing systems encoding catalase mRNA, which, when delivered in vivo, attenuated oxidative stress, reduced neuroinflammation, and partially restored striatal integrity in 6-OHDA models [127]. This strategy highlights the potential of engineered EV-producing systems to improve therapeutic efficiency through localized and sustained delivery.

Overall, MSC-EVs exert multifactorial benefits in PD, targeting inflammation, protein aggregation, oxidative stress and autophagy, while engineered and cargo-enhanced formulations further amplify these effects, offering a modular platform for disease-specific optimization.

### 6.3. Multiple Sclerosis

Given the multifactorial nature of multiple sclerosis pathology, therapeutic strategies capable of simultaneously modulating inflammation, promoting remyelination, and preserving neuronal metabolism are particularly appealing. Although current disease-modifying therapies effectively reduce inflammatory relapses, their impact on progressive neurodegeneration and long-term repair remains limited. In this context, MSC-EVs have gained attention as a cell-free therapeutic strategy due to their ability to modulate immune cell activation, support remyelination, and limit axonal and mitochondrial damage.

In EAE models, MSC-EVs modulate both innate and adaptive immune responses, reducing neuroinflammation through regulation of pro- and anti-inflammatory cytokines and promoting the expansion of regulatory T cells (Tregs). This immunoregulatory shift is associated with improved neurological outcomes, increased generation of oligodendrocytes, elevated myelin basic protein levels and enhanced microglial polarization toward an anti-inflammatory M2 phenotype [163,164]. However, EAE models do not fully recapitulate the complexity and chronicity of human MS.

Dose-dependent effects have also been reported. Low doses of MSC-EVs show limited efficacy, whereas higher concentrations improve clinical outcomes and protect against oligodendroglia degeneration [165]. These effects are partly mediated by suppression of NLRP3 inflammasome activation and downstream neuroinflammatory signaling [166]. Moreover, as in many other neurodegenerative diseases, dysregulation of anti-inflammatory miRNAs contributes to disease progression. In this context, engineered MSC-EVs overexpressing miR-181a-5p reduce pyroptosis and microglial activation, thereby mitigating demyelination in EAE models [167].

Therapeutic efficacy is also influenced by timing of administration. Recent evidence suggests that early delivery of MSC-EVs before symptom onset results in lower clinical score and delayed progression, whereas late-stage administration produces more limited effects [168].

Regarding delivery routes, intranasal administration represents a promising non-invasive strategy due to its proximity to the CNS [55]. However, systemic administration studies have shown that MSC-EVs preferentially accumulate in inflamed lymphoid organs, including lymph nodes, where they interact with macrophages, dendritic cells, and T cells, highlighting a potential dual contribution of biodistribution and immune-cell targeting to their therapeutic activity [169].

Finally, preconditioning strategies further enhance therapeutic outcomes. IFN-γ -primed MSC-EVs reduced demyelination, neuroinflammation, and clinical severity, while increasing Treg populations in both in vitro and in vivo MS models [116]. Notably, evidence also suggests that a single administration of MSC-EVs is insufficient for sustained therapeutic benefit, and repeated dosing might be required to achieve functional improvement, a consideration that is likely relevant across neurodegenerative conditions.

### 6.4. Expanding the Therapeutic Frontier of MSC-EVs: ALS and Traumatic Injuries of the Central and Peripheral Nervous System

#### 6.4.1. Amyotrophic Lateral Sclerosis

MSC-EVs have been extensively investigated as therapeutic tools for neuroinflammation, not only in the major pathologies discussed above, but also in a broader spectrum of diseases. In the context of ALS, following the limited success of direct MSC transplantation approaches [170], MSC-EVs have emerged as a compelling alternative strategy. Adipose tissue-derived MSC-EVs have demonstrated the ability to polarize microglial cells toward an anti-inflammatory phenotype and reduce their metabolic activity, while concurrently improving motor performance and downregulating the NF-κB signaling pathway, both in vitro and in vivo [171,172]. Furthermore, MSC-EVs primed with IFN-γ exhibit a cargo enriched in specific miRNAs (in particular miR-467f and miR-466q) that attenuate the release of pro-inflammatory cytokines by primary microglia derived from ALS mouse models [117]. Notably, these two miRNAs exert a broader neuroprotective effect, rescuing the astrocytic phenotype by suppressing the expression of inflammatory mediators, enhancing the activity of the antioxidant enzyme NQO1, and collectively promoting a shift toward the neuroprotective A2 phenotype [137].

In contrast, EVs derived from muscle cells of ALS patients have been shown to exert neurotoxic effects on motor neurons. Compared to vesicles obtained from healthy donors, their administration induces neuronal cell death accompanied by disruption of neurite integrity, suggesting that the paracrine activity of these pathological vesicles may operate as a self-amplifying mechanism that contributes to disease progression [173].

#### 6.4.2. Traumatic Injuries of the Central and Peripheral Nervous System

In mouse models of Traumatic Brain Injury (TBI), a single dose of MSC-EVs has been shown to restore neurogenesis to levels comparable to naïve controls, an effect accompanied by significant upregulation of both pre- and post-synaptic proteins and increased BDNF expression during both the acute and chronic phases [174]. Additionally, MSC-EVs mitigate the long-term damage induced by glutamate excitotoxicity in astrocyte-neuron co-culture models, highlighting their potential to attenuate post-TBI neurological sequelae [175]. Their therapeutic efficacy can be further enhanced through engineering strategies, such as loading with miRNA-expressing lentiviral vectors or using ultra-small paramagnetic nanoparticles for site-specific targeting, leading to reduced neuroinflammation and enhanced brain plasticity [171].

Similarly, MSC-EVs have demonstrated significant promise in Spinal Cord Injury (SCI). As detailed in previous sections (see Section 3.1 and Section 3.3), preconditioning strategies, such as hypoxia or melatonin priming, potentiate their ability to promote an A1-to-A2 astrocyte phenotypic shift and stabilize NRF2 antioxidant signaling, ultimately facilitating motor recovery and limiting secondary tissue damage [87,103]. Specifically, the enrichment of miR-146a-5p and miR-21 in these vesicles has been shown to target neurotoxic astrocytes and promote recovery via the JAK2/STAT3 pathway, while also stimulating local angiogenesis and reducing apoptosis [104].

MSC-EVs have demonstrated remarkable neuroprotective effects in ischemic brain injury. Wang and colleagues showed that MSC-derived EVs significantly reduce infarct volume and improve neurological outcomes after transient cerebral ischemia in mice. Mechanistically, these EVs exert their therapeutic effects by modulating leukocyte response, with a specific impact on neutrophil infiltration and activation in the ischemic brain. By limiting neutrophil-mediated inflammation and preventing secondary tissue damage, MSC-EVs preserve neuronal integrity and promote functional recovery. These findings highlight the potential of MSC-EVs as cell-free therapeutic agents for CNS disorders characterized by neuroinflammation and ischemic injury [176].

MSC-EVs have also shown therapeutic potential in peripheral nervous system (PNS) injuries. Experimental studies indicate that MSC-EVs can promote axonal regeneration, enhance Schwann cell survival and proliferation, modulate local inflammatory responses, and improve functional recovery following peripheral nerve damage. Notably, NGF-preconditioned MSC-EVs have been reported to enhance axonal regrowth and support Schwann cell function, further highlighting the regenerative potential of engineered EV-based approaches [135,177]. These effects, supported by recent evidence [178], are mediated by the transfer of bioactive cargo, including growth factors, proteins, and regulatory RNAs involved in nerve repair and remyelination. Although evidence remains predominantly preclinical, these findings suggest that MSC-EVs may represent a promising therapeutic strategy for both central and peripheral nervous system injuries.

Given the importance of mitochondrial dysfunction in peripheral nerve degeneration and regeneration, mito-EVs may represent a promising future therapeutic avenue. However, evidence in peripheral nerve injury models remains limited and further studies are required to clarify their potential role.

Collectively, these findings underscore the capacity of MSC-EVs to target multiple pathological mechanisms across both central and peripheral nervous system injuries, reinforcing their potential as versatile therapeutic agents.

## 7. Conclusions

Neuroinflammation is now widely recognized as a key driver of neurodegenerative disease progression, yet translating this knowledge into effective therapies remains a major challenge. In this context, MSC-EVs offer a promising cell-free strategy, with well-documented immunomodulatory and neuroprotective effects in preclinical models. Nevertheless, the current body of evidence remains heterogeneous and often lacks standardization, limiting reproducibility and cross-study comparability.

The emerging concept of mito-EVs adds an additional layer of complexity as well as therapeutic opportunity.

Mito-EVs are EVs enriched in mitochondrial components, including mitochondrial DNA, respiratory chain proteins, and antioxidants, and have been shown to transfer functional mitochondria to recipient cells, restoring bioenergetics and reducing oxidative stress. Their potential role in modulating bioenergetics and inflammatory signaling is particularly intriguing, although their functional relevance is still incompletely defined. 

Experimental studies have demonstrated that mito-EVs can attenuate mitochondrial damage, suppress inflammation, and promote mitochondrial biogenesis in injured tissues, ranging from chronic neurodegenerative environments to acute traumatic injuries of the CNS. Notably, their dual potential to either dampen or propagate pathological signals raises relevant safety concerns that need to be carefully addressed.

Preconditioning strategies, including hypoxic, inflammatory, pharmacological and genetic approaches, represent powerful tools to enhance the therapeutic efficacy of MSC-EVs by reshaping their molecular cargo. Evidence suggests that preconditioning can increase the release of mito-EVs and enrich their mitochondrial cargo, thereby potentiating their neuroprotective and immunomodulatory effects. These approaches enable a degree of functional tailoring of EVs for specific pathological contexts, thereby improving their immunomodulatory and neuroprotective potential.

Nevertheless, several key challenges must still be addressed before clinical translation of MSC-EVs and mito-EVs can be achieved. These include the need for standardized isolation and characterization protocols; scalable production methods; accurate definition of EV cargo composition and a deeper understanding of their biodistribution, safety, and long-term effects in vivo. In particular, more data are needed on the quantity and quality of mito-EVs released under different preconditioning conditions, their specific mitochondrial cargo, and their functional impact on neuroinflammation and neurodegeneration in vivo.

Overall, MSC-EVs and mito-EVs represent an attractive therapeutic avenue for neurodegenerative disorders, but their successful translation into disease-modifying strategies will depend on rigorous standardization, deeper mechanistic insight, and well-designed clinical studies.

## Data Availability

No new data were created or analyzed in this study. Data sharing is not applicable to this article.
